# Perceived helpfulness of treatment for generalized anxiety disorder: a World Mental Health Surveys report

**DOI:** 10.1186/s12888-021-03363-3

**Published:** 2021-08-09

**Authors:** Dan J. Stein, Alan E. Kazdin, Ayelet Meron Ruscio, Wai Tat Chiu, Nancy A. Sampson, Hannah N. Ziobrowski, Sergio Aguilar-Gaxiola, Ali Al-Hamzawi, Jordi Alonso, Yasmin Altwaijri, Ronny Bruffaerts, Brendan Bunting, Giovanni de Girolamo, Peter de Jonge, Louisa Degenhardt, Oye Gureje, Josep Maria Haro, Meredith G. Harris, Aimee Karam, Elie G. Karam, Viviane Kovess-Masfety, Sing Lee, Maria Elena Medina-Mora, Jacek Moskalewicz, Fernando Navarro-Mateu, Daisuke Nishi, José Posada-Villa, Kate M. Scott, Maria Carmen Viana, Daniel V. Vigo, Miguel Xavier, Zahari Zarkov, Ronald C. Kessler, on behalf of the WHO World Mental Health Survey collaborators, Sergio Aguilar-Gaxiola, Ali Al-Hamzawi, Mohammed Salih Al-Kaisy, Jordi Alonso, Yasmin A. Altwaijri, Laura Helena Andrade, Lukoye Atwoli, Corina Benjet, Guilherme Borges, Evelyn J. Bromet, Ronny Bruffaerts, Brendan Bunting, Jose Miguel Caldas-de-Almeida, Graça Cardoso, Somnath Chatterji, Alfredo H. Cia, Louisa Degenhardt, Koen Demyttenaere, Silvia Florescu, Giovanni de Girolamo, Oye Gureje, Josep Maria Haro, Meredith G. Harris, Hristo Hinkov, Chi-yi Hu, Peter de Jonge, Aimee Nasser Karam, Elie G. Karam, Norito Kawakami, Ronald C. Kessler, Andrzej Kiejna, Viviane Kovess-Masfety, Sing Lee, Jean-Pierre Lepine, John J. McGrath, Maria Elena Medina-Mora, Zeina Mneimneh, Jacek Moskalewicz, Fernando Navarro-Mateu, Marina Piazza, Jose Posada-Villa, Kate M. Scott, Tim Slade, Juan Carlos Stagnaro, Dan J. Stein, Margreet ten Have, Yolanda Torres, Maria Carmen Viana, Daniel V. Vigo, Harvey Whiteford, David R. Williams, Bogdan Wojtyniak

**Affiliations:** 1 Department of Psychiatry & Mental Health and South African Medical Council Research Unit on Risk and Resilience in Mental Disorders, University of Cape Town and Groote Schuur Hospital, Cape Town, South Africa; 2 Department of Psychology, Yale University, New Haven, CT USA; 3 Department of Psychology, University of Pennsylvania, Philadelphia, PA USA; 4 Department of Health Care Policy, Harvard Medical School, Boston, MA USA; 5 Center for Reducing Health Disparities, UC Davis Health System, Sacramento, California, USA; 6 College of Medicine, Al-Qadisiya University, Diwaniya governorate, Al Diwaniyah, Iraq; 7 Health Services Research Unit, IMIM-Hospital del Mar Medical Research Institute, Barcelona, Spain; 8 CIBER en Epidemiología y Salud Pública (CIBERESP), Madrid, Spain; 9 Pompeu Fabra University (UPF), Barcelona, Spain; 10 Epidemiology Section, King Faisal Specialist Hospital and Research Center, Riyadh, Saudi Arabia; 11 Universitair Psychiatrisch Centrum - Katholieke Universiteit Leuven (UPC-KUL), Campus Gasthuisberg, Leuven, Belgium; 12 School of Psychology, Ulster University, Londonderry, UK; 13 IRCCS Istituto Centro San Giovanni di Dio Fatebenefratelli, Brescia, Italy; 14 Department of Developmental Psychology, University of Groningen, Groningen, Netherlands; 15 Interdisciplinary Center Psychopathology and Emotion Regulation, University Medical Center Groningen, Groningen, Netherlands; 16 National Drug and Alcohol Research Centre, University of New South Wales, Sydney, Australia; 17 Department of Psychiatry, University College Hospital, Ibadan, Nigeria; 18 Parc Sanitari Sant Joan de Déu, CIBERSAM, Universitat de Barcelona, Sant Boi de Llobregat, Barcelona, Spain; 19 School of Public Health, The University of Queensland, Herston, QLD 4006 Australia; 20 Queensland Centre for Mental Health Research, The Park Centre for Mental Health, Wacol, QLD 4072 Australia; 21 Institute for Development, Research, Advocacy & Applied Care (IDRAAC), Beirut, Lebanon; 22 Department of Psychiatry and Clinical Psychology, St George Hospital University Medical Center, Beirut, Lebanon; 23 Faculty of Medicine, Balamand University, Beirut, Lebanon; 24 Ecole des Hautes Etudes en Santé Publique (EHESP), EA 4057, Paris Descartes University, Paris, France; 25 Department of Psychiatry, Chinese University of Hong Kong, Tai Po, Hong Kong; 26 National Institute of Psychiatry-Ramón de la Fuente Muñiz, Mexico City, Mexico; 27 Institute of Psychiatry and Neurology, Warsaw, Poland; 28 UDIF-SM, Servicio Murciano de Salud; IMIB-Arrixaca; CIBERESP-Murcia, Región de Murcia, Murcia, Spain; 29 Department of Mental Health, Graduate School of Medicine, The University of Tokyo, Tokyo, Japan; 30 National Center of Neurology and Psychiatry, Tokyo, Japan; 31 Colegio Mayor de Cundinamarca University, Faculty of Social Sciences, Bogota, Colombia; 32 Department of Psychological Medicine, University of Otago, Dunedin, Otago New Zealand; 33 Department of Social Medicine, Postgraduate Program in Public Health, Federal University of Espírito Santo, Vitoria, Brazil; 34 Department of Psychiatry, University of British Columbia, Vancouver, BC Canada; 35 Department of Global Health and Social Medicine, Harvard Medical School, Boston, MA USA; 36 Lisbon Institute of Global Mental Health and Chronic Diseases Research Center (CEDOC), NOVA Medical School-Faculdade de Ciências Médicas, Universidade Nova de Lisboa, Lisbon, Portugal; 37 Department of Mental Health, National Center of Public Health and Analyses, Sofia, Bulgaria

**Keywords:** Generalized anxiety disorder, Pathways to treatment, Patient-centered outcomes, Treatment helpfulness

## Abstract

**Background:**

Treatment guidelines for generalized anxiety disorder (GAD) are based on a relatively small number of randomized controlled trials and do not consider patient-centered perceptions of treatment helpfulness. We investigated the prevalence and predictors of patient-reported treatment helpfulness for DSM-5 GAD and its two main treatment pathways: encounter-level treatment helpfulness and persistence in help-seeking after prior unhelpful treatment.

**Methods:**

Data came from community epidemiologic surveys in 23 countries in the WHO World Mental Health surveys. DSM-5 GAD was assessed with the fully structured WHO Composite International Diagnostic Interview Version 3.0. Respondents with a history of GAD were asked whether they ever received treatment and, if so, whether they ever considered this treatment helpful. Number of professionals seen before obtaining helpful treatment was also assessed. Parallel survival models estimated probability and predictors of a given treatment being perceived as helpful and of persisting in help-seeking after prior unhelpful treatment.

**Results:**

The overall prevalence rate of GAD was 4.5%, with lower prevalence in low/middle-income countries (2.8%) than high-income countries (5.3%); 34.6% of respondents with lifetime GAD reported ever obtaining treatment for their GAD, with lower proportions in low/middle-income countries (19.2%) than high-income countries (38.4%); 3) 70% of those who received treatment perceived the treatment to be helpful, with prevalence comparable in low/middle-income countries and high-income countries. Survival analysis suggested that virtually all patients would have obtained helpful treatment if they had persisted in help-seeking with up to 10 professionals. However, we estimated that only 29.7% of patients would have persisted that long. Obtaining helpful treatment at the person-level was associated with treatment type, comorbid panic/agoraphobia, and childhood adversities, but most of these predictors were important because they predicted persistence rather than encounter-level treatment helpfulness.

**Conclusions:**

The majority of individuals with GAD do not receive treatment. Most of those who receive treatment regard it as helpful, but receiving helpful treatment typically requires persistence in help-seeking. Future research should focus on ensuring that helpfulness is included as part of the evaluation. Clinicians need to emphasize the importance of persistence to patients beginning treatment.

**Supplementary Information:**

The online version contains supplementary material available at 10.1186/s12888-021-03363-3.

## Background

Generalized Anxiety Disorder (GAD) encompasses a variety of symptoms including excessive worrying, restlessness, irritability, difficulties in concentration, and constantly feeling on edge. Among the anxiety disorders, GAD is perhaps the least well researched or understood in part because of multiple revisions of the diagnostic criteria, consideration of the diagnosis as a “wastebasket” category when other anxiety diagnoses could not made, and because worrying was assumed a part of everyday life and associated with minimal impairment. However, GAD is now known now to be a significant disorder with high prevalence and significant impairment and disability [1, 2]. Worldwide an estimated 3.7% of individuals will have GAD in their lifetime [1]. The role and quality of life impairments of GAD are comparable in magnitude to those of major depression and greater than those associated with substance abuse disorders [3].

Randomized controlled trials (RCTs) have identified effective pharmacological and psychosocial treatments [4–7]. However, results from RCTs do not necessarily translate into real-world settings and are largely restricted to high-income countries (HICs) with little global evaluation of the effects of treatment [8–10]. Moreover, RCTs have focused on symptom reduction as the critical and sometimes the sole outcome. Yet, symptom reduction does not necessarily equate with functional improvement, quality of life, or feelings on the part of the patient that they have been helped [11, 12].

Perceived helpfulness of treatment is a key construct that may capture whether patients achieve personally meaningful goals through treatment. Helpfulness is important in its own right, but also has a critical role in treatment in that perceived helpfulness relates directly to treatment adherence as well as to critical processes that span diverse forms psychotherapy (e.g., the therapeutic alliance, critical incidents during treatment, openness of the therapist) [13–16]. Helpfulness as an outcome of treatment has received little attention in GAD trials [17]. The extent to which helpfulness is achieved with treatment and whether this is achieved initially as the patient traverses different treatments remain to be evaluated. Such research might help identify unmet patient needs that can be targeted through policy and service responses [14, 18, 19]. Patient-centered research on perceived GAD treatment helpfulness may also inform treatment guidelines for GAD, which are currently based on a relatively small number of RCTs.

The likelihood of a help-seeking individual ever obtaining helpful treatment is a joint function of two treatment pathways: 1) the probability that a given treatment provider will be helpful, and 2) the probability that a patient will persist in help-seeking after prior unhelpful treatment encounters. Research on these processes for depression and posttraumatic stress disorder found that the majority of patients who persisted after previous unhelpful treatments eventually obtained helpful treatments, but that only a minority persisted in help-seeking after more than a few unsuccessful treatment encounters [20, 21]. It is unknown whether these patterns also are true for GAD. Decomposing treatment pathways for GAD would answer this question and might also reveal modifiable predictors of GAD treatment helpfulness and persistence that could be the focus of treatment quality improvement initiatives.

The present study evaluated the helpfulness of treatment encounters and predictors and pathways leading to helpfulness in national community epidemiological samples that included respondents with a history of GAD who sought treatment for their GAD. The data come from the World Mental Health (WMH) Survey Initiative [22]. This is a coordinated series of general population surveys under the auspices of the World Health Organization. Data were collected in 23 countries of varying income levels. This data set provides a unique opportunity to evaluate helpfulness internationally and to investigate predictors and pathways of perceived treatment response in countries varying in income levels.

## Methods

### Samples

The World Health Organization’s (WHO) World Mental Health (WMH) surveys are a coordinated set of community epidemiological surveys administered to probability samples of the non-institutionalized household population in countries throughout the world (https://www.hcp.med.harvard.edu/wmh/) [23]. Data for the current report came from 26 WMH surveys carried out in 23 countries – 9 in countries classified by the World Bank as low/middle-income (Brazil, Bulgaria [separate surveys carried out in 2002 and 2016], Colombia, Colombia [Medellin], Iraq, Lebanon, Mexico, and Peru) and 17 in countries classified as HICs (Argentina, Australia, Belgium, France, Germany, Israel, Italy, Japan, the Kingdom of Saudi Arabia, the Netherlands, New Zealand, Northern Ireland, Poland, Portugal, Spain, Spain [Murcia], and the United States). Response rates ranged from 45.9% (France) to 97.2% (Colombia [Medellin]) and averaged 67.4% across surveys (see eTable 1 for detailed survey characteristics).

The interview schedule was developed in English and translated into other languages using a standardized WHO translation, back-translation, and harmonization protocol [24]. Interviews were administered face-to-face in respondents’ homes after obtaining informed consent using procedures approved by local Institutional Review Boards. To reduce respondent burden, interviews were administered in two parts. Part I was administered to all respondents and assessed core DSM-IV mental disorders. Part II assessed additional disorders and correlates and was administered to all respondents who met lifetime criteria for any Part I disorder and a probability subsample of other Part I respondents [25].

### Measures

#### Generalized anxiety disorder (GAD)

Lifetime history of GAD was assessed with the fully structured WHO Composite International Diagnostic Interview (CIDI) Version 3.0 [23]. In an evaluation carried out in conjunction with the US WMH survey [26], GAD diagnoses based on the CIDI had good concordance with diagnoses based on blinded clinical reassessments with the Structured Clinical Interview for DSM-IV (SCID) [27]. A clinical reappraisal study in other WMH surveys, although not evaluating GAD in isolation, found good concordance between diagnoses based on the CIDI and SCID for any 12-month anxiety disorder including GAD [28]. Consistent with previous studies that modified the CIDI GAD algorithm [1, 26, 29], we generated DSM-5 GAD diagnoses by removing the DSM-IV hierarchical exclusion of a GAD diagnosis when symptoms occur exclusively during a mood disorder [30]. Age of onset (AOO) of GAD was assessed using probing methods demonstrated to improve dating accuracy [31].

#### Perceived helpfulness of treatment for GAD

Respondents who met lifetime criteria for GAD were asked whether they had ever “talk [ed] to a medical doctor or other professional about their worry or anxiety” and, if so, how old they were the first time they did so. “Other professionals” were defined broadly to include “psychologists, counselors, spiritual advisors, herbalists, acupuncturists, and other healing professionals.” Respondents who had ever spoken to a professional about their GAD were asked whether they ever received treatment they “considered helpful or effective” (*emphasis in original)*. If so, they were asked how many professionals they ever talked to about their worry or anxiety “up to and including the first time [they] ever got helpful treatment”. Respondents who said they never received helpful treatment were asked how many professionals they ever talked to about their worry or anxiety.

#### Predictors of obtaining helpful treatment for GAD

In addition to age of onset of GAD (continuous), we considered 5 classes of predictors of helpful treatment: *Socio-demographic characteristics* included sex, marital status (currently married, never married, or previously married) at the time of first treatment, and education (in quartiles defined by within-country distributions) at the time of first treatment. *Lifetime comorbid conditions* included other lifetime anxiety disorders (including panic disorder or agoraphobia with/ without panic disorder, post-traumatic stress disorder, specific phobia, and social phobia), major depressive disorder, broadly defined bipolar spectrum disorder [32], alcohol and/ or drug abuse, and alcohol and/ or drug dependence but not abuse. Comorbid conditions were restricted to disorders with an age-of-onset prior to the age at which the respondent first sought treatment for GAD. All comorbid conditions were assessed with the CIDI. *Treatment type* was defined as a cross-classification of (i) whether the respondent reported receiving medication, psychotherapy, or both, as of the age of first GAD treatment; and (ii) the types of treatment providers seen as of that age. Types of providers included mental health specialists (psychiatrist, psychiatric nurse, psychologist, psychiatric social worker, mental health counselor), primary care providers, human services providers (social worker or counselor in a social services agency, spiritual advisor), and complementary-alternative medicine (other type of healer or self-help group). *Treatment timing* included a continuous variable for length of delay in years between age of onset of GAD and age of initially seeking treatment, and a dichotomous measure for whether the respondent’s first attempt to seek treatment occurred before or after the year 2000. The year 2000 was the typical midpoint between the start of observations and survey field dates. *Childhood adversities* included retrospective reports of significant stressors experienced during childhood, including family dysfunction (physical or sexual abuse, neglect, parental mental disorder, parental substance use disorder, parental criminal behavior, and family violence) and other adversities (parental death, parental divorce, other loss of a parent, physical illness, and economic adversity).

### Analysis methods

The sample was limited to respondents with a history of DSM-5 GAD treatment who sought treatment for the disorder at some time in their life. Cases were limited to those who first sought GAD treatment in 1990 or later to reduce the potential effects of recall bias. To investigate the two pathways of helpful treatment separately, we used discrete-event survival analysis to calculate the conditional and cumulative probabilities of: (i) obtaining helpful treatment after seeing between 1 and 10 professionals; and (ii) persisting in help-seeking after obtaining prior unhelpful treatment [33]. We followed respondents up through 10 professionals in the total sample and in HICs because this was the last number where at least *n* = 30 respondents received treatment. However, in low/middle-income countries (LMICs), we followed respondents only up through 3 professionals seen because this was the last number where at least n = 30 respondents received treatment.

We then carried out parallel survival analyses of the predictors of these two decomposed, encounter-level outcomes using standard discrete-time methods and a logistic link function [34], followed by a person-level model of overall probability of ever obtaining helpful treatment regardless of number of professionals seen (composite outcome). This allowed us to investigate predictors of obtaining helpful treatment at the person level and to investigate the extent to which these predictors were important because they predicted differential helpfulness at the encounter level, differential probability of persisting after earlier unhelpful treatments, or both.

We also investigated possible interactions of each significant person-level predictor with country income group and historical time (beginning treatment in 1990–1999 vs. 2000+) in an effort to examine the generalizability of the findings. Because the WMH sample design used weighting and clustering in all countries, all statistical analyses were carried out using the Taylor series linearization method [35], a design-based method implemented in the SAS 9.4 program [36]. Logistic regression coefficients and +/− 2 of their design-based standard errors were exponentiated to create odds-ratios (ORs) and 95% confidence intervals (CIs) (with odds ratios less than 1 indicating lower likelihood, and odds ratios greater than 1 indicating greater likelihood, of the relevant association). Significance of sets of coefficients was evaluated with Wald χ^2^ tests based on design-corrected coefficient variance-covariance matrices. Statistical significance was evaluated using two-sided, design-based .05 level tests.

## Results

### GAD prevalence, treatment, and perceived helpfulness of treatment

Lifetime prevalence (SE) of GAD was 2.8% (0.1) in LMICs, 5.3% (0.1) in HICs, and 4.5% (0.1) across all WMH surveys (Table 1). Among respondents with lifetime GAD, approximately one-third reported ever obtaining GAD treatment (34.6% [0.8]) and 70.0% (1.4) of those who obtained GAD treatment perceived the treatment to be helpful. Respondents with GAD in LMICs were significantly less likely than those in HICs to obtain GAD treatment (19.2% vs. 38.4%; χ^2^_1_ = 63.6, *p* < 0.001), but probability of treatment being perceived as helpful did not differ significantly by country income level (62.8% vs. 70.9%; χ^2^_1_ = 2.7, *p* = 0.099).

**Table 1:** Lifetime prevalence of DSM-5 generalized anxiety disorder (GAD), proportion of respondents with lifetime GAD who obtained treatment, and proportion of treated respondents who perceived treatment as helpful

	Total sample			Respondents with lifetime GAD			Respondents who obtained treatment for GAD		
	Prevalence of lifetime GAD			Proportion who obtained treatment^**a**^			Proportion who perceived treatment as helpful^**b**^		
	%	(SE)	(n)	%	(SE)	(n)	%	(SE)	(n)
**I. Low/middle-income countries**									
Sao Paulo, Brazil	5.1	(0.4)	(5037)	23.6	(3.5)	(280)	71.4	(7.4)	(63)
Bulgaria	2.2	(0.2)	(6826)	16.8	(3.2)	(157)	69.4	(10.0)	(27)
Colombia	1.9	(0.3)	(4426)	15.8	(4.6)	(84)	45.0	(17.4)	(15)
Medellin, Colombia	3.8	(0.5)	(3261)	17.9	(3.4)	(127)	73.7	(9.8)	(31)
Iraq	5.0	(0.6)	(4332)	22.2	(5.5)	(220)	52.5	(13.8)	(33)
Lebanon	2.3	(0.3)	(2857)	12.4	(4.7)	(71)	38.1	(19.1)	(9)
Mexico	1.1	(0.2)	(5782)	8.5	(3.8)	(78)	28.4	(17.6)	(8)
Peru	1.0	(0.1)	(3930)	23.0	(8.3)	(40)	82.1	(8.5)	(10)
All	2.8	(0.1)	(36,451)	19.2	(1.8)	(1057)	62.8	(4.9)	(196)
χ^2^_7_	242.3*			8.4			11.2		
**II. High-income countries**									
Argentina	5.2	(0.6)	(3927)	34.7	(6.0)	(224)	85.1	(6.0)	(70)
Australia	8.0	(0.4)	(8463)	49.0	(2.5)	(713)	76.7	(3.0)	(326)
Belgium	3.2	(0.8)	(1043)	35.6	(12.5)	(62)	33.9	(18.5)	(20)
France	6.0	(0.7)	(1436)	33.2	(4.4)	(159)	71.1	(7.7)	(62)
Germany	1.7	(0.4)	(1323)	34.2	(8.7)	(48)	80.5	(13.3)	(19)
Israel	4.4	(0.3)	(4859)	36.9	(3.4)	(216)	44.6	(5.9)	(76)
Italy	1.9	(0.3)	(1779)	24.3	(4.8)	(78)	89.1	(6.2)	(25)
Japan	2.6	(0.3)	(4129)	30.9	(4.8)	(105)	67.5	(10.2)	(30)
Netherlands	3.6	(0.4)	(1094)	46.4	(6.3)	(95)	88.3	(5.3)	(45)
New Zealand	7.9	(0.3)	(12,790)	41.2	(1.9)	(1084)	68.0	(2.8)	(411)
Northern Ireland	6.4	(0.4)	(4340)	39.6	(2.3)	(334)	70.2	(4.9)	(131)
Poland	0.9	(0.1)	(10,081)	37.8	(5.0)	(90)	73.7	(7.4)	(33)
Portugal	6.1	(0.5)	(3849)	41.3	(2.7)	(269)	73.4	(5.7)	(104)
Saudi Arabia	2.3	(0.4)	(3638)	27.5	(9.1)	(81)	56.7	(17.7)	(23)
Spain	1.9	(0.2)	(2121)	36.3	(5.0)	(114)	90.1	(3.5)	(41)
Murcia, Spain	7.0	(0.9)	(2621)	37.5	(4.6)	(193)	76.4	(9.4)	(80)
United States	7.8	(0.3)	(9282)	28.1	(2.3)	(752)	69.5	(3.2)	(205)
All	5.3	(0.1)	(76,775)	38.4	(0.9)	(4617)	70.9	(1.4)	(1701)
	**%**	**(SE)**	**(n)**	**%**	**(SE)**	**(n)**	**%**	**(SE)**	**(n)**
χ^2^_16_	731.6*			53.7*			54.1*		
**III. Pooled countries**									
All countries	4.5	(0.1)	(113,226)	34.6	(0.8)	(5674)	70.0	(1.4)	(1897)
χ^2^_24_	1276.3*			150.4*			67.0*		
Low/middle**-**income countries vs. high-income countries									
χ^2^_1_	206.6*			63.6*			2.7		

Abbreviations. *GAD* generalized anxiety disorder; SE, standard error

*Significant at the .05 level, two-sided test

^a^Cases are based on three conditions: (i) Respondents obtained GAD treatment; (ii) Year of first GAD treatment ≥1990; and (iii) Age at onset of GAD ≤ Year of first GAD treatment

^b^Cases are based on four conditions: (i) Respondents obtained GAD treatment; (ii) Year of first GAD treatment ≥1990; and (iii) Age at onset of GAD ≤ Year of first GAD treatment; and (iv) Respondents obtained helpful treatment

### Helpful GAD treatment across professionals seen

Across all countries, 26.7% (1.0) of respondents who received treatment said they were helped by the first professional they saw (Table 2, Part I). The conditional probability of obtaining helpful treatment from a second professional seen after an initial unhelpful treatment was 36.6%. Conditional probabilities of obtaining helpful treatment generally declined after subsequent professionals seen but projected cumulative probabilities of obtaining helpful treatment rose from 26.7% after the first professional seen to 53.5% among respondents who persisted in seeing a second professional and to 96.9% among those who persisted in seeing up to 10 professionals after prior unhelpful treatments (Table 2, Part II). These cumulative probabilities were broadly similar for LMICs and HICs up through three professionals seen (66.9% vs. 68.9%), after which the number of remaining respondents in LMICs became too small for analysis (eTable 2).

**Table 2:** Conditional and cumulative probabilities of obtaining helpful treatment for generalized anxiety disorder after each professional seen, among respondents with lifetime DSM-5 generalized anxiety disorder who obtained treatment

	I. Conditional probabilities of obtaining helpful treatment			II. Cumulative probabilities of obtaining helpful treatment (***n*** = 1897)	
Number of professionals seen until the respondent obtained helpful treatment	%	(SE)	(n)	%	(SE)
1	26.7	(1.0)	(1897)	26.7	(1.0)
2	36.6	(1.7)	(1070)	53.5	(1.6)
3	33.1	(2.4)	(585)	68.9	(1.7)
4	29.0	(3.4)	(335)	77.9	(1.5)
5	25.2	(3.1)	(213)	83.5	(1.4)
6	33.1	(5.1)	(136)	89.0	(1.2)
7	16.4	(4.7)	(88)	90.8	(1.2)
8	16.6	(3.7)	(72)	92.3	(1.1)
9	3.5	(3.4)	(59)	92.6	(1.1)
10	57.9	(7.2)	(58)	96.9	(0.7)

Abbreviations. *SE* standard error

### Persistence with help-seeking for GAD following treatment failure

Across all countries, 77.3% (1.1) of respondents persisted in seeing a second professional after initial unhelpful GAD treatment (Table 3). This proportion was higher in HICs (79.6% [1.2]) than LMICs (55.8% [3.5]). Conditional probabilities of help-seeking persistence remained quite high (81.9–100.0%) in the total sample up through 10 professionals seen. Unlike the situation with cumulative probabilities of obtaining helpful treatment, which, by definition, either remains the same or rises as the number of professionals seen increases, the cumulative probability of help-seeking persistence either remains the same or decreases as the number of professionals seen increases. In the total sample, the cumulative probability of persistence through 10 professionals was 29.7%. We were able to compare respondents in LMICs to those in high income countries through four professionals seen, where the cumulative probability of persistence was lower in LMICs (43.2% [5.9]) than in high income countries (57.5% [2.5]) (eTable 3).

**Table 3:** Conditional and cumulative probabilities of persistence in help-seeking after previous unhelpful treatment, among respondents with lifetime DSM-5 generalized anxiety disorder who obtained treatment

	I. Conditional probabilities of persistence in help-seeking			II. Cumulative probabilities of persistence in help-seeking (***n*** = 1373)	
Number of professionals seen after not being helped previously	%	(SE)	(n)	%	(SE)
2	77.3	(1.1)	(1373)	77.3	(1.1)
3	83.4	(1.5)	(696)	64.4	(1.9)
4	87.1	(1.8)	(385)	56.1	(2.3)
5	83.9	(2.1)	(248)	47.1	(2.4)
6	83.8	(2.8)	(157)	39.5	(2.6)
7	81.9	(4.9)	(102)	32.3	(2.9)
8	94.6	(1.9)	(77)	30.6	(2.9)
9	97.0	(0.4)	(61)	29.7	(2.9)
10	100.0	(0.0)	(58)	29.7	(2.9)

Abbreviations. *SE* standard error

### Predictors of obtaining helpful treatment for GAD

We examined predictors of each respondent ever obtaining helpful GAD treatment regardless of number of professionals seen (Model 1; Table 4), then examined predictors separately for the two pathways to helpful treatment: obtaining helpful treatment from a given professional (Model 2; Table 4) and persisting in help-seeking after prior unhelpful treatment (Model 3; Table 4). We focus on the significant predictors at the person-level (Model 1; Table 4) and examine how the results in the decomposed models help explain these person-level associations. Due to high comorbidity between disorders and the potential for multicollinearity, we evaluated associations with comorbid disorders in separate univariate and then multivariate models. Only comorbid disorders that significantly predicted obtaining helpful treatment in the multivariate models were included in Models 1–3 as individual predictors (eTable 4).

**Table 4:** Multivariable analysis of predictors of obtaining helpful treatment (person-level composite outcome) and of the decomposed encounter-level outcomes of helpful treatment and persistence, among people with lifetime DSM-5 generalized anxiety disorder who obtained treatment

	Composite outcome				Decomposed outcomes							
	Model 1: Person-level relative-odds of obtaining helpful treatment (regardless of the number of professionals seen)				Model 2: Encounter-level relative-odds of obtaining helpful treatment from a given professional (pooled across professionals seen)				Model 3: Encounter-level relative-odds of the respondent persisting in help-seeking after prior unhelpful treatment (pooled across all unhelpful treatments)			
	**Prevalence**		**Multivariate**		**Prevalence**		**Multivariate**		**Prevalence**		**Multivariate**	
	**Mean/%**	**(SE)**	**OR**	**(95% CI)**	**Mean/%**	**(SE)**	**OR**	**(95% CI)**	**Mean/%**	**(SE)**	**OR**	**(95% CI)**
Age of GAD onset (years)	29.4	(0.3)	1.01	(1.00–1.02)	28.4	(0.4)	1.02*	(1.01–1.02)	27.7	(0.4)	0.99	(0.98–1.00)
χ^2^_1_	2.63				22.41*				1.48			
Female	67.3	(1.2)	1.18	(0.90–1.54)	66.8	(1.6)	1.21*	(1.03–1.41)	66.2	(1.8)	1.08	(0.85–1.39)
χ^2^_1_	1.47				5.51*				0.42			
Marital Status												
Never married	36.9	(1.2)	1.06	(0.80–1.42)	36.6	(1.5)	1.18*	(1.01–1.38)	36.2	(1.7)	0.85	(0.65–1.10)
Previously married	22.9	(1.2)	0.99	(0.71–1.36)	24.0	(1.6)	0.88	(0.73–1.06)	24.6	(1.8)	1.08	(0.80–1.46)
Currently married	40.2	(1.2)	1.00	–	39.4	(1.5)	1.00	–	39.2	(1.7)	1.00	–
χ^2^_2_	0.21				9.66*				2.03			
Education												
Low	13.7	(0.8)	0.63*	(0.42–0.95)	15.1	(1.1)	0.62*	(0.48–0.80)	16.1	(1.4)	0.92	(0.66–1.28)
Low-average	23.7	(1.2)	0.79	(0.57–1.09)	23.3	(1.4)	0.95	(0.77–1.17)	23.2	(1.6)	0.86	(0.62–1.18)
High-average	31.9	(1.2)	0.80	(0.58–1.10)	30.6	(1.5)	0.96	(0.81–1.15)	30.0	(1.7)	0.80	(0.58–1.10)
Student	10.4	(0.8)	0.59*	(0.36–0.99)	12.0	(1.2)	0.81	(0.59–1.11)	12.7	(1.5)	0.62*	(0.39–0.99)
High	20.2	(0.9)	1.00	–	19.0	(1.1)	1.00	–	18.0	(1.2)	1.00	–
χ^2^_4_	7.74				17.37*				4.43			
Treatment delay (years)^a^	6.4	(0.3)	0.99	(0.98–1.00)	6.1	(0.3)	1.01*	(1.00–1.02)	6.2	(0.4)	0.98*	(0.96–0.99)
χ^2^_1_	1.95				4.24*				12.44*			
Started GAD treatment > = 2000 (vs. 1990–1999)	52.6	(1.2)	0.78	(0.60–1.01)	45.5	(1.4)	1.22*	(1.06–1.40)	43.1	(1.5)	0.61*	(0.48–0.76)
χ^2^_1_	3.66				7.25*				18.38*			
Treatment type^b^												
Mental health specialist + psychotherapy	55.3	(1.2)	0.83	(0.62–1.12)	60.3	(1.6)	0.73*	(0.59–0.91)	61.9	(1.8)	1.33	(0.98–1.81)
Mental health specialist + medication	55.7	(1.4)	1.46*	(1.05–2.02)	65.8	(1.6)	0.76*	(0.62–0.93)	67.8	(1.8)	2.00*	(1.50–2.67)
General medical	74.1	(1.1)	0.88	(0.63–1.22)	79.4	(1.2)	0.60*	(0.50–0.72)	81.5	(1.3)	1.56*	(1.14–2.14)
Complementary/alternative medicine	20.4	(1.0)	1.38	(0.97–1.97)	27.9	(1.5)	0.73*	(0.63–0.84)	30.1	(1.7)	1.99*	(1.43–2.77)
Human services	16.6	(0.9)	1.00	–	21.0	(1.4)	1.00	–	22.6	(1.6)	1.00	–
χ^2^_4_	12.27*				50.39*				32.32*			
Exactly 2 or more of the above	67.5	(1.1)	1.30	(0.89–1.89)	77.7	(1.1)	1.01	(0.75–1.35)	80.1	(1.2)	1.49*	(1.03–2.15)
χ^2^_1_	1.80				0.00				4.56*			
χ^2^_5_	27.34*				77.21*				131.57*			
Comorbidity												
Number of comorbid disorders^c^	1.7	(0.0)	1.06	(0.92–1.21)	1.9	(0.0)	0.98	(0.91–1.06)	1.9	(0.0)	1.10	(0.98–1.24)
χ^2^_1_	0.67				0.20				2.56			
Panic disorder or agoraphobia with/without panic	19.5	(1.0)	1.79*	(1.22–2.62)	24.8	(1.7)	0.93	(0.78–1.12)	25.8	(1.9)	2.12*	(1.53–2.94)
χ^2^_1_	8.89*				0.57				20.15*			
Major depressive disorder	51.3	(1.3)	1.21	(0.91–1.61)	51.4	(1.4)	1.26*	(1.07–1.49)	50.6	(1.6)	0.91	(0.69–1.20)
χ^2^_1_	1.64				7.83*				0.47			
Bipolar disorder	10.9	(0.8)	1.40	(0.89–2.21)	12.9	(1.2)	1.18	(0.93–1.50)	13.3	(1.3)	1.15	(0.76–1.75)
χ^2^_1_	2.12				1.77				0.44			
χ^2^_4_	24.46*				10.08*				42.86*			
Childhood adversities												
Family dysfunction^d^	31.4	(1.1)	0.75*	(0.58–0.96)	31.8	(1.4)	0.93	(0.78–1.10)	32.4	(1.5)	0.73*	(0.57–0.93)
Other^e^	18.0	(0.9)	1.47*	(1.09–1.99)	17.8	(1.1)	1.13	(0.95–1.34)	17.5	(1.2)	1.51*	(1.10–2.07)
χ^2^_2_	9.73*				2.84				12.06*			
Global χ^2^_21_	88.45*				181.41*				260.66*			

Abbreviations: *CI* confidence interval; *GAD* generalized anxiety disorder; *OR* odds ratio; *SE* standard error

*Significant at .05 level, two-sided test

^a^Treatment delay (years) = Age at first GAD treatment – Age at onset of GAD

^b^Treatment providers: mental health specialists (psychiatrist, psychiatric nurse, psychologist, psychiatric social worker, mental health counselor), primary care providers, human services providers (social worker or counselor in a social services agency, spiritual advisor), and complementary/alternative medicine (other type of healer or self-help group)

^c^Comorbid disorders include: panic disorder or agoraphobia with/without panic disorder, post-traumatic stress disorder, specific phobia, social phobia, major depressive disorder, bipolar disorder, alcohol and/or drug abuse, and alcohol or drug dependence but not abuse

^d^Family dysfunction includes: physical abuse, sexual abuse, neglect, parental mental disorder, parental substance use disorder, parental criminal behavior, and family violence

^e^Other childhood adversities include: parental death, parental divorce, other loss of a parent, physical illness, and economic adversity

At the person-level, significant predictors of obtaining helpful treatment were treatment type (χ^2^_4_ = 12.3, *p* = 0.015), comorbid panic/agoraphobia (χ^2^_1_ = 8.9, *p* = 0.003), and childhood adversities (χ^2^_2_ = 9.7, *p* = 0.008). The association with treatment type was because respondents who received treatment from a mental health specialist in combination with medication had significantly increased relative-odds of obtaining helpful treatment than those who received treatment in the human services sector (the reference category; OR:1.46; 95% CI: 1.05, 2.02). Decomposition showed that this person-level association of treatment from a mental health specialist in combination with the outcome was due to lower relative-odds of encounter-level helpfulness (OR: 0.76; 95% CI: 0.62, 0.93), but higher relative-odds of treatment persistence (OR: 2.00; 95% CI: 1.50, 2.67). Comorbid panic/agoraphobia was associated at the person-level with having significantly increased relative-odds of obtaining helpful treatment (OR: 1.79; 95% CI: 1.22, 2.62) due to increased relative-odds of treatment persistence (OR: 2.12; 95% CI: 1.53, 2.94).

Childhood adversities were also important predictors of treatment helpfulness, but unexpectedly, the pattern of associations was different for the two classes of adversities. A history of family dysfunction was significantly associated with reduced relative-odds of obtaining helpful treatment at the person-level (OR: 0.75; 95% CI: 0.58, 0.96), whereas a history of other childhood adversities was significantly associated with increased relative-odds of obtaining helpful treatment (OR: 1.47; 95% CI: 1.09, 1.99). Decomposition showed that the person-level association of family dysfunction with the outcome was due to significantly reduced relative-odds of treatment persistence (OR: 0.73; 95% CI: 0.57, 0.93), whereas the person-level association other childhood adversities with the outcome was due to significantly increased relative-odds of treatment persistence (OR: 1.51; 95% CI: 1.10, 2.07). It is noteworthy that the zero- Pearson correlation between the two CA measures is too low (r = 0.23) to create an opposite-sign pattern as a methodological artifact. Consistent with this observation, the same opposite-sign pattern was also observed in models where only one of the two CA measures was included.

Although the omnibus χ^2^ tests for treatment timing and treatment delay were not significant, there were significantly increased relative-odds of both predictors with significantly decreased relative-odds of with persistence in help-seeking after an initial unhelpful treatment, were found for both beginning treatment in more recent years (2000 or later) and longer treatment delays in obtaining helpful treatment from a given professional, resulting in nonsignificant associations at the person-level.

We found significant interactions of treatment type and childhood adversities with country income level (eTable 5). These interactions were due to the predictors being more strongly associated with person-level treatment helpfulness in LMICs than HICs (eTables 6 and 7), but the significant associations were based on such small numbers of cases that substantive interpretation is hazardous. Significant interactions were also found between treatment type and historical time (eTable 8). These were due to mental health specialist and psychotherapy treatment and general medical treatment both having increased relative-odds of person-level treatment helpfulness only during the years 1990–1999 and receiving treatment from 2+ types of professionals having increased relative-odds of person-level treatment helpfulness only during the years 2000+ (eTables 9 and 10). Again, though, these interactions were based on relatively small numbers of cases and should be interpreted with caution.

## Discussion

The main findings of the study are as follows. First, only about one-third of people with GAD reported ever obtaining treatment, with a lower proportion in LMICs than HICs (19.2% vs. 38.4%). Second, 70% of those who received treatment perceived the treatment to be helpful. This did not vary by country income level. Third, persistence in help-seeking was required to obtain helpful treatment, as only about one-fourth of patients were helped by the first professional they saw and about half by the first two professionals. Projections from our survival models suggest that up to 10 professionals might be needed to have a 90% probability of being helped, but that only 29.7% of patients would persist that long in the face of repeated unhelpful treatment encounters. Fourth, only relatively modest predictors were found of obtaining helpful treatment at the person-level, most of which were important because they predicted persistence rather than encounter-level treatment helpfulness.

It is encouraging that the large majority (70.0%) of respondents with lifetime DSM-5 GAD who sought treatment eventually obtained treatment they considered helpful. This means that the majority of patients persisted up to 3–4 professions in the face of initial unhelpful treatment. This is a lower persistence rate than found in a parallel analysis of specific phobia [37], but a higher persistence rate than found in parallel analyses of patients with major depression [20] and PTSD [21], possibly because depressive and PTSD symptoms are more likely than anxiety symptoms to lead to discouragement in help-seeking. Even so, we estimated than only 22.7% of GAD patients would persist in help-seeking to a point where they had near certainty of receiving helpful treatment.

Our data on the predictors of helpfulness are useful in beginning to delineate pathways. Patients who received treatment from a mental health specialist in combination with medication had significantly increased relative-odds of obtaining helpful treatment at the person-level than those who received treatment in the human services sector. This was due to lower relative-odds of encounter-level helpfulness, but higher relative-odds of treatment persistence. Patients who receive medication in addition to seeing a mental health specialist may have been more severe than other patients, resulting in lower relative-odds of encounter-level helpfulness due to the severity of their illness, but also more motivation to persist in help-seeking because of that high severity. Likewise, panic/agoraphobia was also associated with having significantly increased relative-odds of obtaining helpful treatment at the person-level due to increased relative-odds of treatment persistence. These individuals may have more chronic and impairing courses of illness [38] which may motivate them to persist in help-seeking.

The relationship between childhood adversity and treatment helpfulness was less straightforward. Family dysfunction was associated with lower relative-odds of obtaining helpful treatment due to lower relative-odds of treatment persistence. In contrast, other childhood adversities were associated with greater relative-odds of obtaining helpful treatment due to increased relative-odds of treatment persistence. One possible explanation for this difference is that the family dysfunction category included violent and traumatic forms of adversity, whereas the other childhood adversities category did not [39]. If this finding is replicated in other studies, future research should focus on why traumatic childhood events are associated with lower persistence in help-seeking, which could inform treatment guidelines.

Important limitations of this study should be noted. First, there was limited information about the precise nature of the interventions that respondents received and no information about sequencing of treatments across types of providers. Moreover, the treatments were not randomized nor evaluated in relation to quality of delivery (treatment integrity) or compliance on the part of the patient (adherence). Consequently, the relation of critical dimensions of treatment to helpfulness could not be discerned. Second, the sample was limited to respondents with onset of GAD treatment after 1990. Recall may have been biased and influenced evaluations of the treatments and helpfulness [40]. It is unclear whether these limitations would lead to upward or downward bias in estimates of treatment effectiveness at the encounter level or patient level. Despite these limitations, to our knowledge this is the first study of perceived helpfulness of treatment of GAD. A strength of the study is including large sample representing multiple countries and with the ability to evaluate commonalities and differences among low and middle income and high- income countries.

RCTs are clearly required to determine the efficacy and effectiveness of GAD treatments [4–7]. Although our data do not fill this need, they are important because they address issues that RCTs cannot. Specifically, RCTs typically focus on short-term effects (e.g., 3 months), assess mainly symptomatic changes, and exclude many people who might have more complicated disorders, such as those with psychiatric comorbidities but who would benefit from treatment [8]. Our study, in comparison, looked at a broad and representative sample without these exclusionary criteria and included information on how patients view their treatment. We would encourage the assessment of treatment helpfulness in clinical trials because it is distinguishable from symptomatic change. One can readily conceive of patients showing similar or identical changes on standardized symptom measures but in fact profiting in different degrees from treatment in their everyday lives and hence in their views of how much they have been helped [41].

Our findings suggest that treatment guidelines should not only encourage evidence-based interventions, but also should emphasize the value of treatment persistence. Our data do not allow us to study new treatments from the same provider. The persistence we examined was across providers. It should be noted, though, that evidence is clear in showing that patients can also be helped by new treatments from the same provider [42]. Further work is needed to expand GAD care to address treatment motivations and expectations and to determine the extent to which interventions to improve GAD treatment quality and persistence can improve outcomes.

## Supplementary Information

**Additional file 1: Table 1.** WMH sample characteristics by World Bank income categories^a^. **Table 2**. Conditional and cumulative probabilities of obtaining helpful treatment for generalized anxiety disorder after each professional seen, among respondents with lifetime DSM-5 generalized anxiety disorder who obtained treatment in low/middle-income and high-income countries. **Table 3.** Conditional and cumulative probabilities of persistence in help-seeking after previous unhelpful treatment, among respondents with lifetime DSM-5 generalized anxiety disorder who obtained treatment in low/middle-income and high-income countries. **Table 4.** Predictors of obtaining helpful treatment (person-level), among people with lifetime DSM-5 generalized anxiety disorder who obtained treatment. **Table 5.** Interactions between main effects and country income group to predict obtaining helpful treatment (person-level composite outcome) and the decomposed encounter-level outcomes of helpful treatment and persistence, among people with lifetime DSM-5 generalized anxiety disorder who obtained treatment. **Table 6.** (Low/middle-income countries): Predictors of obtaining helpful treatment (person-level composite outcome) and of the decomposed encounter-level outcomes of helpful treatment and persistence, among people with lifetime DSM-5 generalized anxiety disorder who obtained treatment. **Table 7.** (High-income countries): Predictors of obtaining helpful treatment (person-level composite outcome) and of the decomposed encounter-level outcomes of helpful treatment and persistence, among people with lifetime DSM-5 generalized anxiety disorder who obtained treatment. **Table 8.** Interactions between main effects and historical time to predict obtaining helpfulness of treatment (person-level composite outcome) and the decomposed encounter-level outcomes of helpful treatment and persistence, among people with lifetime DSM-5 generalized anxiety disorder who obtained treatment. **Table 9.** (Started GAD treatment in 2000 or later): Predictors of obtaining helpful treatment (person-level composite outcome) and of the decomposed encounter-level outcomes of helpful treatment and persistence, among people with lifetime DSM-5 generalized anxiety disorder who obtained treatment. **Table 10.** (Started GAD treatment 1990 to 1999): Predictors of obtaining helpful treatment (person-level composite outcome) and of the decomposed encounter-level outcomes of helpful treatment and persistence, among people with lifetime DSM-5 generalized anxiety disorder who obtained treatment.

## Data Availability

Access to the cross-national World Mental Health (WMH) data is governed by the organizations funding and responsible for survey data collection in each country. These organizations made data available to the WMH consortium through restricted data sharing agreements that do not allow us to release the data to third parties. The exception is that the U.S. data are available for secondary analysis via the Inter-University Consortium for Political and Social Research (ICPSR), http://www.icpsr.umich.edu/icpsrweb/ICPSR/series/00527.

## Abbreviations

CIDI: Composite International diagnostic interview
GAD: Generalized anxiety disorder
RCT: Randomized control trial
WHO: World health organization
WMH: World mental health
