# Supplementary material for: Perceived helpfulness of treatment for generalized anxiety disorder: a World Mental Health Surveys report
Source: BMC Psychiatry. 2021 Aug 9;21:392. doi: 10.1186/s12888-021-03363-3 (PMC8351147; doi:10.1186/s12888-021-03363-3)
Supplement: Supplementary file 1 — Additional file 1: Table 1. WMH sample characteristics by World Bank income categoriesa. Table 2. Conditional and cumulative probabilities of obtaining helpful treatment for generalized anxiety disorder after each professional seen, among respondents with lifetime DSM-5 generalized anxiety disorder who obtained treatment in low/middle-income and high-income countries. Table 3. Conditional and cumulative probabilities of persistence in help-seeking after previous unhelpful treatment, among respondents with lifetime DSM-5 generalized anxiety disorder who obtained treatment in low/middle-income and high-income countries. Table 4. Predictors of obtaining helpful treatment (person-level), among people with lifetime DSM-5 generalized anxiety disorder who obtained treatment. Table 5. Interactions between main effects and country income group to predict obtaining helpful treatment (person-level composite outcome) and the decomposed encounter-level outcomes of helpful treatment and persistence, among people with lifetime DSM-5 generalized anxiety disorder who obtained treatment. Table 6. (Low/middle-income countries): Predictors of obtaining helpful treatment (person-level composite outcome) and of the decomposed encounter-level outcomes of helpful treatment and persistence, among people with lifetime DSM-5 generalized anxiety disorder who obtained treatment. Table 7. (High-income countries): Predictors of obtaining helpful treatment (person-level composite outcome) and of the decomposed encounter-level outcomes of helpful treatment and persistence, among people with lifetime DSM-5 generalized anxiety disorder who obtained treatment. Table 8. Interactions between main effects and historical time to predict obtaining helpfulness of treatment (person-level composite outcome) and the decomposed encounter-level outcomes of helpful treatment and persistence, among people with lifetime DSM-5 generalized anxiety disorder who obtained treatment. Table 9. (Started GAD treatment in 20 [file 12888_2021_3363_MOESM1_ESM.docx]

| **eTable 1. WMH sample characteristics by World Bank income categories^a^** | | | | | | | | |
| --- | --- | --- | --- | --- | --- | --- | --- | --- |
|  | | | | | | | | |
|  |  |  |  | **Sample size** | | | |  |
| **Country by income category** | **Survey^b^** | **Sample characteristics^c^** | **Field dates** | **Age range** | **Part I** | **Part II** | **Part II and age ≤ 44^d^** | **Response rate^e^** |
| **I. Low/middle-income countries** | | |  |  |  |  |  |  |
| Brazil - São Paulo | São Paulo Megacity | São Paulo metropolitan area. | 2005-8 | 18-93 | 5,037 | 2,942 | -- | 81.3 |
| Bulgaria | NSHS | Nationally representative. | 2002-6 | 18-98 | 5,318 | 2,233 | 741 | 72.0 |
| Bulgaria 2 | NNSHS-2 | Nationally representative. | 2016-17 | 18-91 | 1,508 | 578 | -- | 61.0 |
| Colombia | NSMH | All urban areas of the country (approximately 73% of the total national population). | 2003 | 18-65 | 4,426 | 2,381 | 1,731 | 87.7 |
| Colombia – Medellín^f^ | MMHHS | Medellín metropolitan area. | 2011-12 | 19-65 | 3,261 | 1,673 | -- | 97.2 |
| Iraq | IMHS | Nationally representative. | 2006-7 | 18-96 | 4,332 | 4,332 | -- | 95.2 |
| Lebanon | LEBANON | Nationally representative. | 2002-3 | 18-94 | 2,857 | 1,031 | 595 | 70.0 |
| Mexico | M-NCS | All urban areas of the country (approximately 75% of the total national population). | 2001-2 | 18-65 | 5,782 | 2,362 | 1,736 | 76.6 |
| Peru | EMSMP | Five urban areas of the country (approximately 38% of the total national population). | 2004-5 | 18-65 | 3,930 | 1,801 | 1,287 | 90.2 |
| **TOTAL** |  |  |  |  | (36,451) | (19,333) | (6,090) | 81.0 |
| **II. High-income countries** | | |  |  |  |  |  |  |
| Argentina | AMHES | Eight largest urban areas of the country (approximately 50% of the total national population). | 2015 | 18-98 | 3,927 | 2,116 | -- | 77.3 |
| Australia^g^ | NSMHWB | Nationally representative. | 2007 | 18-85 | 8,463 | 8,463 | -- | 60.0 |
| Belgium | ESEMeD | Nationally representative. The sample was selected from a national register of Belgian residents. | 2001-2 | 18-95 | 2,419 | 1,043 | 486 | 50.6 |
| France | ESEMeD | Nationally representative. The sample was selected from a national list of households with listed telephone numbers. | 2001-2 | 18-97 | 2,894 | 1,436 | 727 | 45.9 |
| Germany | ESEMeD | Nationally representative. | 2002-3 | 19-95 | 3,555 | 1,323 | 621 | 57.8 |
| Israel | NHS | Nationally representative. | 2003-4 | 21-98 | 4,859 | 4,859 | -- | 72.6 |
| Italy | ESEMeD | Nationally representative. The sample was selected from municipality resident registries. | 2001-2 | 18-100 | 4,712 | 1,779 | 853 | 71.3 |
| Japan | WMHJ 2002-6 | Eleven metropolitan areas. | 2002-6 | 20-98 | 4,129 | 1,682 | -- | 55.1 |
| Netherlands | ESEMeD | Nationally representative. The sample was selected from municipal postal registries. | 2002-3 | 18-95 | 2,372 | 1,094 | 516 | 56.4 |
| New Zealand^g^ | NZMHS | Nationally representative. | 2004-5 | 18-98 | 12,790 | 7,312 | -- | 73.3 |
| Northern Ireland | NISHS | Nationally representative. | 2005-8 | 18-97 | 4,340 | 1,986 | -- | 68.4 |
| Poland | EZOP | Nationally representative. | 2010-11 | 18-65 | 10,081 | 4,000 | 2,276 | 50.4 |
| Portugal | NMHS | Nationally representative. | 2008-9 | 18-81 | 3,849 | 2,060 | 1,070 | 57.3 |
| Saudi Arabia^g^ | SNMHS | Nationally representative | 2013-16 | 18-65 | 3,638 | 1,793 | -- | 61.0 |
| Spain | ESEMeD | Nationally representative. | 2001-2 | 18-98 | 5,473 | 2,121 | 960 | 78.6 |
| Spain – Murcia | PEGASUS- Murcia | Murcia region. Regionally representative. | 2010-12 | 18-96 | 2,621 | 1,459 | -- | 67.4 |
| **eTable 1 continued. WMH sample characteristics by World Bank income categories^a^** | | | | | | | | |
|  |  |  |  | **Sample size** | | | |  |
| **Country by income category** | **Survey^b^** | **Sample characteristics^c^** | **Field dates** | **Age range** | **Part I** | **Part II** | **Part II and age ≤ 44^d^** | **Response rate^e^** |
| United States | NCS-R | Nationally representative. | 2001-3 | 18-99 | 9,282 | 5,692 | 3,197 | 70.9 |
| **TOTAL** |  |  |  |  | (89,404) | (50,218) | (10.706) | 63.0 |
| **III. TOTAL** |  |  |  |  | (125, 855) | (69,551) | (16,796) | 67.4 |
|  | | | | | | | | |

^a^The World Bank (2012) Data. Accessed May 12, 2012 at: http://data.worldbank.org/country. Some of the WMH countries have moved into new income categories since the surveys were conducted. The income groupings above reflect the status of each country at the time of data collection. The current income category of each country is available at the preceding URL.

^b^NSHS (Bulgaria National Survey of Health and Stress); NSMH (The Colombian National Study of Mental Health); MMHHS (Medellín Mental Health Household Study); IMHS (Iraq Mental Health Survey); LEBANON (Lebanese Evaluation of the Burden of Ailments and Needs of the Nation); M-NCS (The Mexico National Comorbidity Survey); EMSMP (La Encuesta Mundial de Salud Mental en el Peru); AMHES (Argentina Mental Health Epidemiologic Survey); NSMHWB (National Survey of Mental Health and Wellbeing); ESMeD (The European Study Of The Epidemiology Of Mental Disorders); WMHJ2002-2006 (World Mental Health Japan Survey); NZMHS (New Zealand Mental Health Survey); NISHS (Northern Ireland Study of Health and Stress); EZOP (Epidemiology of Mental Disorders and Access to Care Survey); NMHS (Portugal National Mental Health Survey); SNMHS (Saudi National Mental Health Survey); PEGASUS-Murcia (Psychiatric Enquiry to General Population in Southeast Spain-Murcia);NCS-R (The US National Comorbidity Survey Replication).

^c^Most World Mental Health surveys are based on stratified multistage clustered area probability household samples in which samples of areas equivalent to counties or municipalities in the US were selected in the first stage followed by one or more subsequent stages of geographic sampling (e.g., towns within counties, blocks within towns, households within blocks) to arrive at a sample of households, in each of which a listing of household members was created and one or two people were selected from this listing to be interviewed. No substitution was allowed when the originally sampled household resident could not be interviewed. These household samples were selected from Census area data in all countries other than France (where telephone directories were used to select households) and the Netherlands (where postal registries were used to select households). Several WMH surveys (Belgium, Germany, Italy, Poland, Spain-Murcia) used municipal, country resident, or universal health-care registries to select respondents without listing households. The Japanese sample is the only totally un-clustered sample, with households randomly selected in each of the 11 metropolitan areas and one random respondent selected in each sample household. 18 of the 26 surveys are based on nationally representative household samples.

^d^Argentina, Australia, Brazil, Bulgaria 2 (2016-17), Colombia-Medellin, Iraq, Israel, Japan, New Zealand, Northern Ireland, Saudi Arabia and Spain-Murcia did not have an age restricted Part 2 sample. All other surveys were age restricted to ≤ 44.

^e^The response rate is calculated as the ratio of the number of households in which an interview was completed to the number of households originally sampled, excluding from the denominator households known not to be eligible either because of being vacant at the time of initial contact or because the residents were unable to speak the designated languages of the survey. The weighted average response rate is 67.4%.

^f^Colombia mover from the “lower and lower-middle income” to the “upper-middle income” category between 2003 (when the Colombian National Study of Mental Health was conducted) and 2010 (when the Medellin Mental Health Household Study was conducted, hence Colombia’s appearance in both income categories. For more information, please see footnote *a*.

^g^For the purposes of cross-national comparisons we limit the sample to those 18+.

| **eTable 2. Conditional and cumulative probabilities of obtaining helpful treatment for generalized anxiety disorder after each professional seen, among respondents with lifetime DSM-5 generalized anxiety disorder who obtained treatment in low/middle-income and high-income countries** | | | | | | | | | | | | | |
| --- | --- | --- | --- | --- | --- | --- | --- | --- | --- | --- | --- | --- | --- |
|  | | | | | | | | | | | | | |
|  | **I. Conditional probabilities of obtaining helpful treatment** | | | | | | |  | **II. Cumulative probabilities of obtaining helpful treatment** | | | | |
| **Number of professionals seen until the respondent obtained helpful treatment** | **Low/middle-income countries** | | |  | **High-income countries** | | |  | **Low/middle-income countries (n=196)** | |  | **High-income countries**  **(n=1,701)** | |
|  | **%** | **(SE)** | **(n)** |  | **%** | **(SE)** | **(n)** |  | **%** | **(SE)** |  | **%** | **(SE)** |
| 1 | 34.2 | (2.9) | (196) |  | 25.7 | (1.1) | (1,701) |  | 34.2 | (2.9) |  | 25.7 | (1.1) |
| 2 | 34.2 | (5.9) | (77) |  | 36.8 | (1.8) | (993) |  | 56.7 | (5.6) |  | 53.0 | (1.7) |
| 3 | 23.5 | (5.4) | (43) |  | 33.8 | (2.6) | (542) |  | 66.9 | (5.4) |  | 68.9 | (1.7) |
| 4 |  |  |  |  | 27.4 | (3.3) | (307) |  |  |  |  | 77.4 | (1.6) |
| 5 |  |  |  |  | 24.7 | (3.3) | (198) |  |  |  |  | 83.0 | (1.5) |
| 6 |  |  |  |  | 35.1 | (5.3) | (127) |  |  |  |  | 89.0 | (1.3) |
| 7 |  |  |  |  | 15.9 | (5.2) | (79) |  |  |  |  | 90.7 | (1.2) |
| 8 |  |  |  |  | 18.1 | (4.0) | (66) |  |  |  |  | 92.4 | (1.1) |
| 9 |  |  |  |  | 3.9 | (3.8) | (53) |  |  |  |  | 92.7 | (1.1) |
| 10 |  |  |  |  | 63.0 | (7.2) | (52) |  |  |  |  | 97.3 | (0.6) |
|  |  |  |  |  |  |  |  |  |  |  |  |  |  |

Abbreviations. SE, standard error.

| **eTable 3. Conditional and cumulative probabilities of persistence in help-seeking after previous unhelpful treatment, among respondents with lifetime DSM-5 generalized anxiety disorder who obtained treatment in low/middle-income and high-income countries** | | | | | | | | | | | | | |
| --- | --- | --- | --- | --- | --- | --- | --- | --- | --- | --- | --- | --- | --- |
|  | | | | | | | | | | | | | |
|  | **I. Conditional probabilities of persistence in help-seeking** | | | | | | |  | **II. Cumulative probabilities of persistence in help-seeking** | | | | |
| **Number of professionals seen after not being helped previously** | **Low/middle-income countries** | | |  | **High-income countries** | | |  | **Low/middle-income countries (n=130)** | |  | **High-income countries**  **(n=1,243)** | |
|  | **%** | **(SE)** | **(n)** |  | **%** | **(SE)** | **(n)** |  | **%** | **(SE)** |  | **%** | **(SE)** |
| 2 | 55.8 | (3.5) | (130) |  | 79.6 | (1.2) | (1,243) |  | 55.8 | (3.5) |  | 79.6 | (1.2) |
| 3 | 82.5 | (4.5) | (54) |  | 83.5 | (1.5) | (642) |  | 46.0 | (6.0) |  | 66.4 | (1.9) |
| 4 | 93.9 | (2.0) | (31) |  | 86.5 | (1.9) | (354) |  | 43.2 | (5.9) |  | 57.5 | (2.5) |
| 5 |  |  |  |  | 83.5 | (2.3) | (229) |  |  |  |  | 48.0 | (2.6) |
| 6 |  |  |  |  | 84.5 | (2.9) | (146) |  |  |  |  | 40.6 | (2.8) |
| 7 |  |  |  |  | 80.2 | (5.2) | (93) |  |  |  |  | 32.5 | (3.1) |
| 8 |  |  |  |  | 96.6 | (1.8) | (69) |  |  |  |  | 31.4 | (3.1) |
| 9 |  |  |  |  | 96.6 | (0.5) | (55) |  |  |  |  | 30.3 | (3.1) |
| 10 |  |  |  |  | 100.0 | (0.0) | (52) |  |  |  |  | 30.3 | (3.1) |
|  | | | | | | | | | | | | | |

Abbreviations. SE, standard error.

| **eTable 4. Predictors of obtaining helpful treatment (person-level), among people with lifetime DSM-5 generalized anxiety disorder who obtained treatment** | | | | | | | | | | |
| --- | --- | --- | --- | --- | --- | --- | --- | --- | --- | --- |
|  |  | |  |  | |  |  |  |  | |
|  | **Model 1:**  **Person-level relative-odds of obtaining helpful**  **treatment (regardless of the number of professionals seen)** | | | | | | | | | |
|  | **Prevalence** | |  | **Bivariate** | | | |  | **Multivariate** | |
| **Comorbid disorder** | **%** | **(SE)** |  | **OR** | **(95% CI)** |  | **χ ^2^_1_** |  | **OR** | **(95% CI)** |
| Panic disorder or agoraphobia with/without panic disorder | 19.5 | (1.0) |  | 2.07* | (1.54-2.79) |  | 22.83* |  | 1.86* | (1.35-2.57) |
| Specific phobia | 18.1 | (0.8) |  | 1.39* | (1.06-1.84) |  | 5.47* |  | 1.14 | (0.84-1.54) |
| Social phobia | 26.9 | (1.2) |  | 1.48* | (1.12-1.96) |  | 7.50* |  | 1.25 | (0.91-1.72) |
| Posttraumatic stress disorder | 18.4 | (1.0) |  | 1.14 | (0.87-1.50) |  | 0.90 |  | 1.04 | (0.78-1.37) |
| Major depressive disorder | 51.3 | (1.3) |  | 1.31* | (1.05-1.63) |  | 5.87* |  | 1.36* | (1.08-1.71) |
| Bipolar disorder | 10.9 | (0.8) |  | 1.47* | (1.04-2.07) |  | 4.78* |  | 1.55* | (1.07-2.23) |
| Alcohol and/or drug abuse | 20.6 | (1.1) |  | 0.86 | (0.65-1.13) |  | 1.16 |  | 0.79 | (0.59-1.05) |
| Alcohol or drug dependence but not abuse | 2.5 | (0.3) |  | 1.83 | (0.94-3.55) |  | 3.18 |  | 1.19 | (0.61-2.33) |
| χ^2^_8_ for the significance of the set of disorders |  |  |  |  |  |  |  |  | 45.93* | |
| χ^2^_7_ for the significance of differences across the different disorders |  |  |  |  |  |  |  |  | 22.76* | |
|  |  |  |  |  |  |  |  |  |  | |

Abbreviations. CI, confidence interval; OR, odds ratio; SE, standard error.

Note. Models controlled for country dummies.

*Significant at .05 level, two-sided test.

| **eTable 5. Interactions between main effects and country income group to predict obtaining helpful treatment (person-level composite outcome) and the decomposed encounter-level outcomes of helpful treatment and persistence, among people with lifetime DSM-5 generalized anxiety disorder who obtained treatment** | | | | | | | | | | | | | | | | | |
| --- | --- | --- | --- | --- | --- | --- | --- | --- | --- | --- | --- | --- | --- | --- | --- | --- | --- |
|  | | | | | | | | | | | | | | | | | |
|  | **Composite outcome** | | | | |  | **Decomposed outcome** | | | | | | | | | | |
|  | **Model 1:**  **Person-level relative-odds of**  **obtaining helpful**  **treatment**  **(regardless of the number of**  **professionals seen)** | | | | |  | **Model 2:**  **Encounter-level relative-odds of obtaining helpful treatment from**  **a given professional**  **(pooled across professionals seen)** | | | | |  | **Model 3:**  **Encounter-level relative-odds of the respondent persisting in help-seeking after prior unhelpful treatment**  **(pooled across all unhelpful treatments)** | | | | |
|  | **Prevalence** | |  | **Multivariate** | |  | **Prevalence** | |  | **Multivariate** | |  | **Prevalence** | |  | **Multivariate** | |
| **Interaction terms between each predictor and a country income level** | **Mean/%** | **(SE)** |  | **OR** | **(95% CI)** |  | **Mean/%** | **(SE)** |  | **OR** | **(95% CI)** |  | **Mean/%** | **(SE)** |  | **OR** | **(95% CI)** |
| Treatment type^a^ |  |  |  |  |  |  |  |  |  |  |  |  |  |  |  |  |  |
| Mental health specialist + psychotherapy | 50.2 | (1.3) |  | 0.24* | (0.10-0.56) |  | 55.4 | (1.6) |  | 0.82 | (0.35-1.92) |  | 57.3 | (1.8) |  | 0.29* | (0.10-0.80) |
| Mental health specialist + medication | 51.2 | (1.3) |  | 0.09* | (0.04-0.24) |  | 61.4 | (1.5) |  | 0.60 | (0.31-1.18) |  | 63.7 | (1.7) |  | 0.05* | (0.02-0.15) |
| General medical | 69.0 | (1.1) |  | 0.62 | (0.29-1.36) |  | 75.1 | (1.1) |  | 1.12 | (0.61-2.04) |  | 77.1 | (1.2) |  | 0.53 | (0.22-1.27) |
| Complementary/alternative medicine | 18.9 | (1.0) |  | 0.10* | (0.03-0.31) |  | 26.3 | (1.5) |  | 0.72 | (0.27-1.94) |  | 28.6 | (1.7) |  | 0.07* | (0.02-0.24) |
| Human services | 14.2 | (0.9) |  | 1.00 | - |  | 18.9 | (1.3) |  | 1.00 | - |  | 20.6 | (1.5) |  | 1.00 | - |
| χ^2^_4_ | 30.80* | | | | |  | 6.91 | | | | |  | 41.38* | | | | |
| Exactly 2 or more of the above | 62.7 | (1.2) |  | 10.40* | (3.79-28.51) |  | 73.1 | (1.1) |  | 0.70 | (0.25-1.95) |  | 75.8 | (1.3) |  | 66.08* | (18.45-236.65) |
| χ^2^_1_ | 20.70* | | | | |  | 0.47 | | | | |  | 41.46* | | | | |
| χ^2^_5_ | 30.81* | | | | |  | 37.33* | | | | |  | 69.65* | | | | |
| Panic disorder or agoraphobia with/without panic | 17.7 | (1.0) |  | 0.63 | (0.23-1.72) |  | 22.9 | (1.6) |  | 1.32 | (0.80-2.17) |  | 23.8 | (1.8) |  | 0.43 | (0.13-1.36) |
| χ^2^_1_ | 0.82 | | | | |  | 1.16 | | | | |  | 2.07 | | | | |
| Childhood adversities |  |  |  |  |  |  |  |  |  |  |  |  |  |  |  |  |  |
| Family dysfunction^b^ | 26.4 | (1.1) |  | 2.14* | (1.18-3.90) |  | 27.3 | (1.3) |  | 1.40 | (0.88-2.24) |  | 27.7 | (1.5) |  | 1.40 | (0.57-3.43) |
| Other^c^ | 13.9 | (0.8) |  | 0.32* | (0.18-0.59) |  | 14.3 | (1.0) |  | 0.48* | (0.32-0.72) |  | 14.4 | (1.1) |  | 0.46 | (0.21-1.01) |
| χ^2^_2_ | 15.85* | | | | |  | 14.49* | | | | |  | 4.34 | | | | |
| Global χ^2^_8_ | 62.79* | | | | |  | 58.56* | | | | |  | 88.65* | | | | |
|  |  |  |  |  |  |  |  |  |  |  |  |  |  |  |  |  |  |

Abbreviations. CI, confidence interval; GAD, generalized anxiety disorder; OR, odds ratio; SE, standard error.

*Significant at .05 level, two-sided test.

^a^Treatment providers: mental health specialists (psychiatrist, psychiatric nurse, psychologist, psychiatric social worker, mental health counselor), primary care providers, human services providers (social worker

or counselor in a social services agency, spiritual advisor), and complementary/alternative medicine (other type of healer or self-help group).

^b^Family dysfunction includes: physical abuse, sexual abuse, neglect, parental mental disorder, parental substance use disorder, parental criminal behavior, and family violence.

^c^Other childhood adversities include: parental death, parental divorce, other loss of a parent, physical illness, and economic adversity.

| **eTable 6. (Low/middle-income countries): Predictors of obtaining helpful treatment (person-level composite outcome) and of the decomposed encounter-level outcomes of helpful treatment and persistence, among people with lifetime DSM-5 generalized anxiety disorder who obtained treatment** | | | | | | | | | | | | | | | | | |
| --- | --- | --- | --- | --- | --- | --- | --- | --- | --- | --- | --- | --- | --- | --- | --- | --- | --- |
|  | | | | | | | | | | | | | | | | | |
|  | **Composite outcome** | | | | |  | **Decomposed outcomes** | | | | | | | | | | |
|  | **Model 1:**  **Person-level relative-odds of**  **obtaining helpful**  **treatment**  **(regardless of the number of professionals seen)** | | | | |  | **Model 2:**  **Encounter-level relative-odds of obtaining helpful treatment from**  **a given professional**  **(pooled across professionals seen)** | | | | |  | **Model 3:**  **Encounter-level relative-odds of the respondent persisting in help-seeking after prior unhelpful treatment**  **(pooled across all unhelpful treatments)** | | | | |
|  | **Prevalence** | |  | **Multivariate** | |  | **Prevalence** | |  | **Multivariate** | |  | **Prevalence** | |  | **Multivariate** | |
|  | **Mean/%** | **(SE)** |  | **OR** | **(95% CI)** |  | **Mean/%** | **(SE)** |  | **OR** | **(95% CI)** |  | **Mean/%** | **(SE)** |  | **OR** | **(95% CI)** |
| Treatment type^a^ |  |  |  |  |  |  |  |  |  |  |  |  |  |  |  |  |  |
| Mental health specialist + psychotherapy | 46.4 | (2.5) |  | 3.32* | (1.53-7.21) |  | 53.2 | (3.1) |  | 0.97 | (0.44-2.15) |  | 52.2 | (3.6) |  | 3.42* | (1.60-7.30) |
| Mental health specialist + medication | 41.0 | (3.7) |  | 15.23* | (6.18-37.54) |  | 48.1 | (6.7) |  | 1.48 | (0.86-2.53) |  | 46.3 | (7.3) |  | 34.89* | (12.89-94.39) |
| General medical | 46.4 | (3.9) |  | 1.48 | (0.75-2.94) |  | 47.3 | (6.4) |  | 0.62 | (0.35-1.09) |  | 50.4 | (7.4) |  | 2.26* | (1.13-4.53) |
| Complementary/alternative medicine | 13.8 | (2.8) |  | 14.19* | (4.78-42.17) |  | 17.2 | (4.7) |  | 1.10 | (0.42-2.88) |  | 16.4 | (5.1) |  | 26.56* | (7.52-93.88) |
| Human services | 21.8 | (2.5) |  | 1.00 | - |  | 22.2 | (3.9) |  | 1.00 | - |  | 22.8 | (4.7) |  | 1.00 | - |
| χ^2^_4_ | 43.82* | | | | |  | 28.91* | | | | |  | 58.45* | | | | |
| Exactly 2 or more of the above | 43.8 | (2.6) |  | 0.13* | (0.05-0.32) |  | 50.2 | (3.2) |  | 1.09 | (0.44-2.68) |  | 48.7 | (3.4) |  | 0.04* | (0.01-0.09) |
| χ^2^_1_ | 19.20* | | | | |  | 0.03 | | | | |  | 44.21* | | | | |
| χ^2^_5_ | 48.17* | | | | |  | 50.14* | | | | |  | 59.34* | | | | |
| Panic disorder or agoraphobia with/without panic | 16.8 | (3.0) |  | 3.06* | (1.18-7.90) |  | 21.4 | (6.2) |  | 0.81 | (0.52-1.29) |  | 22.1 | (7.3) |  | 3.44* | (1.41-8.37) |
| χ^2^_1_ | 5.34* | | | | |  | 0.77 | | | | |  | 7.38* | | | | |
| Childhood adversities |  |  |  |  |  |  |  |  |  |  |  |  |  |  |  |  |  |
| Family dysfunction^b^ | 46.0 | (2.9) |  | 0.39* | (0.23-0.66) |  | 49.5 | (2.8) |  | 0.66* | (0.44-0.98) |  | 52.6 | (3.0) |  | 0.51 | (0.25-1.04) |
| Other^c^ | 37.4 | (3.7) |  | 3.26* | (1.97-5.38) |  | 38.4 | (5.1) |  | 1.84* | (1.34-2.51) |  | 35.3 | (5.3) |  | 2.22* | (1.37-3.60) |
| χ^2^_2_ | 25.36* | | | | |  | 21.63* | | | | |  | 10.52* | | | | |
| Global χ^2^_8_ | 128.49* | | | | |  | 124.69* | | | | |  | 83.62* | | | | |
|  |  | | | | |  |  | | | | |  |  | | | | |

Abbreviations. CI, confidence interval; GAD, generalized anxiety disorder; OR, odds ratio; SE, standard error.

*Significant at .05 level, two-sided test.

^a^Treatment providers: mental health specialists (psychiatrist, psychiatric nurse, psychologist, psychiatric social worker, mental health counselor), primary care providers, human services providers (social worker or counselor in a social services agency, spiritual advisor), and complementary/alternative medicine (other type of healer or self-help group).

^b^Family dysfunction includes: physical abuse, sexual abuse, neglect, parental mental disorder, parental substance use disorder, parental criminal behavior, and family violence.

^c^Other childhood adversities include: parental death, parental divorce, other loss of a parent, physical illness, and economic adversity.

| **eTable 7. (High-income countries): Predictors of obtaining helpful treatment (person-level composite outcome) and of the decomposed encounter-level outcomes of helpful treatment and persistence, among people with lifetime DSM-5 generalized anxiety disorder who obtained treatment** | | | | | | | | | | | | | | | | | |
| --- | --- | --- | --- | --- | --- | --- | --- | --- | --- | --- | --- | --- | --- | --- | --- | --- | --- |
|  |  |  |  |  |  |  |  |  |  |  |  |  |  |  |  |  |  |
|  | **Composite outcome** | | | | |  | **Decomposed outcomes** | | | | | | | | | | |
|  | **Model 1:**  **Person-level relative-odds of**  **obtaining helpful**  **treatment**  **(regardless of the number of professionals seen)** | | | | |  | **Model 2:**  **Encounter-level relative-odds of obtaining helpful treatment from**  **a given professional**  **(pooled across professionals seen)** | | | | |  | **Model 3:**  **Encounter-level relative-odds of the respondent persisting in help-seeking after prior unhelpful treatment**  **(pooled across all unhelpful treatments)** | | | | |
|  | **Prevalence** | |  | **Multivariate** | |  | **Prevalence** | |  | **Multivariate** | |  | **Prevalence** | |  | **Multivariate** | |
|  | **Mean/%** | **(SE)** |  | **OR** | **(95% CI)** |  | **Mean/%** | **(SE)** |  | **OR** | **(95% CI)** |  | **Mean/%** | **(SE)** |  | **OR** | **(95% CI)** |
| Treatment type^a^ |  |  |  |  |  |  |  |  |  |  |  |  |  |  |  |  |  |
| Mental health specialist + psychotherapy | 56.5 | (1.4) |  | 0.80 | (0.59-1.09) |  | 61.0 | (1.7) |  | 0.71* | (0.56-0.90) |  | 62.8 | (1.9) |  | 1.25 | (0.92-1.70) |
| Mental health specialist + medication | 57.6 | (1.4) |  | 1.42* | (1.03-1.95) |  | 67.6 | (1.6) |  | 0.76* | (0.62-0.93) |  | 69.9 | (1.8) |  | 2.05* | (1.57-2.69) |
| General medical | 77.5 | (1.1) |  | 0.92 | (0.64-1.33) |  | 82.6 | (1.1) |  | 0.64* | (0.53-0.78) |  | 84.6 | (1.1) |  | 1.50* | (1.06-2.14) |
| Complementary/alternative medicine | 21.2 | (1.1) |  | 1.38 | (0.97-1.96) |  | 28.9 | (1.6) |  | 0.72* | (0.63-0.83) |  | 31.4 | (1.8) |  | 2.09* | (1.49-2.92) |
| Human services | 16.0 | (1.0) |  | 1.00 | - |  | 20.9 | (1.5) |  | 1.00 | - |  | 22.6 | (1.6) |  | 1.00 | - |
| χ^2^_4_ | 11.58* | | | | |  | 45.54* | | | | |  | 37.91* | | | | |
| Exactly 2 or more of the above | 70.5 | 1.2) |  | 1.32 | (0.89-1.96) |  | 80.5 | (1.1) |  | 0.90 | (0.65-1.23) |  | 83.2 | (1.3) |  | 1.75* | (1.19-2.57) |
| χ^2^_1_ | 1.95 | | | | |  | 0.47 | | | | |  | 8.07* | | | | |
| χ^2^_5_ | 23.77* | | | | |  | 84.07* | | | | |  | 127.18* | | | | |
| Panic disorder or agoraphobia with/without panic | 19.8 | (1.1) |  | 1.92* | (1.39-2.65) |  | 25.2 | (1.7) |  | 0.92 | (0.78-1.09) |  | 26.1 | (1.9) |  | 2.38* | (1.76-3.23) |
| χ^2^_1_ | 15.74* | | | | |  | 0.87 | | | | |  | 31.39* | | | | |
| Childhood adversities |  |  |  |  |  |  |  |  |  |  |  |  |  |  |  |  |  |
| Family dysfunction^b^ | 29.6 | (1.2) |  | 0.83 | (0.64-1.08) |  | 30.1 | (1.4) |  | 0.93 | (0.78-1.11) |  | 30.4 | (1.6) |  | 0.76* | (0.60-0.97) |
| Other^c^ | 15.6 | (0.9) |  | 1.06 | (0.78-1.45) |  | 15.7 | (1.1) |  | 0.91 | (0.76-1.09) |  | 15.8 | (1.2) |  | 1.19 | (0.85-1.67) |
| χ^2^_2_ | 1.93 | | | | |  | 1.82 | | | | |  | 6.22* | | | | |
| Global χ^2^_8_ | 50.24* | | | | |  | 91.52* | | | | |  | 163.71* | | | | |
|  |  |  |  |  |  |  |  |  |  |  |  |  |  |  |  |  |  |

Abbreviations. CI, confidence interval; GAD, generalized anxiety disorder; OR, odds ratio; SE, standard error.

*Significant at .05 level, two-sided test.

^a^Treatment providers: mental health specialists (psychiatrist, psychiatric nurse, psychologist, psychiatric social worker, mental health counselor), primary care providers, human services providers (social worker or counselor in a social services agency, spiritual advisor), and complementary/alternative medicine (other type of healer or self-help group).

^b^Family dysfunction includes: physical abuse, sexual abuse, neglect, parental mental disorder, parental substance use disorder, parental criminal behavior, and family violence.

^c^Other childhood adversities include: parental death, parental divorce, other loss of a parent, physical illness, and economic adversity.

| **eTable 8. Interactions between main effects and historical time to predict obtaining helpfulness of treatment (person-level composite outcome) and the decomposed encounter-level outcomes of helpful treatment and persistence, among people with lifetime DSM-5 generalized anxiety disorder who obtained treatment** | | | | | | | | | | | | | | | | | |
| --- | --- | --- | --- | --- | --- | --- | --- | --- | --- | --- | --- | --- | --- | --- | --- | --- | --- |
|  |  | |  |  | |  |  | |  |  | |  |  | |  |  | |
|  | **Composite outcome** | | | | |  | **Decomposed outcome** | | | | | | | | | | |
|  | **Model 1:**  **Person-level relative-odds of**  **obtaining helpful**  **treatment**  **(regardless of the number of professionals seen)** | | | | |  | **Model 2:**  **Encounter-level relative-odds of obtaining helpful treatment from**  **a given professional**  **(pooled across professionals seen)** | | | | |  | **Model 3:**  **Encounter-level relative-odds of the respondent persisting in help-seeking after prior unhelpful treatment**  **(pooled across all unhelpful treatments)** | | | | |
|  | **Prevalence** | |  | **Multivariate** | |  | **Prevalence** | |  | **Multivariate** | |  | **Prevalence** | |  | **Multivariate** | |
| **Interaction terms between each predictor and historical time** | **Mean/%** | **(SE)** |  | **OR** | **(95% CI)** |  | **Mean/%** | **(SE)** |  | **OR** | **(95% CI)** |  | **Mean/%** | **(SE)** |  | **OR** | **(95% CI)** |
| Treatment type^a^ |  |  |  |  |  |  |  |  |  |  |  |  |  |  |  |  |  |
| Mental health specialist + psychotherapy | 27.4 | (1.0) |  | 0.40* | (0.21-0.77) |  | 25.0 | (1.3) |  | 0.79 | (0.55-1.11) |  | 24.0 | (1.4) |  | 0.40* | (0.22-0.73) |
| Mental health specialist + medication | 27.3 | (1.1) |  | 0.58 | (0.33-1.02) |  | 27.6 | (1.4) |  | 0.92 | (0.65-1.29) |  | 26.7 | (1.5) |  | 0.56* | (0.34-0.92) |
| General medical | 37.8 | (1.2) |  | 0.39* | (0.21-0.71) |  | 35.9 | (1.4) |  | 0.43* | (0.30-0.61) |  | 35.7 | (1.5) |  | 0.71 | (0.42-1.21) |
| Complementary/alternative medicine | 9.0 | (0.7) |  | 1.86 | (0.91-3.80) |  | 10.9 | (1.1) |  | 1.13 | (0.78-1.61) |  | 11.0 | (1.2) |  | 1.58 | (0.91-2.77) |
| Human services | 7.7 | (0.7) |  | 1.00 | - |  | 8.7 | (1.0) |  | 1.00 | - |  | 9.1 | (1.1) |  | 1.00 | - |
| χ^2^_4_ | 19.55* | | | | |  | 23.32* | | | | |  | 16.00* | | | | |
| Exactly 2 or more of the above | 33.1 | (1.1) |  | 4.21* | (1.93-9.16) |  | 33.5 | (1.4) |  | 1.53 | (0.91-2.60) |  | 32.6 | (1.6) |  | 4.10* | (2.05-8.19) |
| χ^2^_1_ | 13.07* | | | | |  | 2.53 | | | | |  | 15.97* | | | | |
| χ^2^_5_ | 27.50* | | | | |  | 23.53* | | | | |  | 22.73* | | | | |
| Panic disorder or agoraphobia with/without panic | 10.3 | (0.7) |  | 0.93 | (0.50-1.72) |  | 11.5 | (1.2) |  | 0.96 | (0.69-1.34) |  | 11.4 | (1.3) |  | 0.92 | (0.53-1.61) |
| χ^2^_1_ | 0.06 | | | | |  | 0.06 | | | | |  | 0.08 | | | | |
| Childhood adversities |  |  |  |  |  |  |  |  |  |  |  |  |  |  |  |  |  |
| Family dysfunction^b^ | 16.1 | (0.9) |  | 0.70 | (0.44-1.11) |  | 13.6 | (0.9) |  | 0.81 | (0.59-1.11) |  | 13.1 | (0.9) |  | 1.12 | (0.72-1.73) |
| Other^c^ | 8.7 | (0.7) |  | 1.24 | (0.71-2.18) |  | 7.3 | (0.7) |  | 1.14 | (0.81-1.61) |  | 6.6 | (0.7) |  | 1.04 | (0.58-1.87) |
| χ^2^_2_ | 2.81 | | | | |  | 2.01 | | | | |  | 0.28 | | | | |
| Global χ^2^_8_ | 30.04* | | | | |  | 25.56* | | | | |  | 23.39* | | | | |
|  |  |  |  |  |  |  |  |  |  |  |  |  |  |  |  |  |  |

Abbreviations. CI, confidence interval; GAD, generalized anxiety disorder; OR, odds ratio; SE, standard error.

*Significant at .05 level, two-sided test.

^a^Treatment providers: mental health specialists (psychiatrist, psychiatric nurse, psychologist, psychiatric social worker, mental health counselor), primary care providers, human services providers (social worker

or counselor in a social services agency, spiritual advisor), and complementary/alternative medicine (other type of healer or self-help group).

^b^Family dysfunction includes: physical abuse, sexual abuse, neglect, parental mental disorder, parental substance use disorder, parental criminal behavior, and family violence.

^c^Other childhood adversities include: parental death, parental divorce, other loss of a parent, physical illness, and economic adversity.

| **eTable 9. (Started GAD treatment in 2000 or later): Predictors of obtaining helpful treatment (person-level composite outcome) and of the decomposed encounter-level outcomes of helpful treatment and persistence, among people with lifetime DSM-5 generalized anxiety disorder who obtained treatment** | | | | | | | | | | | | | | | | | |
| --- | --- | --- | --- | --- | --- | --- | --- | --- | --- | --- | --- | --- | --- | --- | --- | --- | --- |
|  |  |  |  |  |  |  |  |  |  |  |  |  |  |  |  |  |  |
|  | **Composite outcome** | | | | |  | **Decomposed outcomes** | | | | | | | | | | |
|  | **Model 1:**  **Person-level relative-odds of**  **obtaining helpful**  **treatment**  **(regardless of the number of professionals seen)** | | | | |  | **Model 2:**  **Encounter-level relative-odds of obtaining helpful treatment from**  **a given professional**  **(pooled across professionals seen)** | | | | |  | **Model 3:**  **Encounter-level relative-odds of the respondent persisting in help-seeking after prior unhelpful treatment**  **(pooled across all unhelpful treatments)** | | | | |
|  | **Prevalence** | |  | **Multivariate** | |  | **Prevalence** | |  | **Multivariate** | |  | **Prevalence** | |  | **Multivariate** | |
|  | **Mean/%** | **(SE)** |  | **OR** | **(95% CI)** |  | **Mean/%** | **(SE)** |  | **OR** | **(95% CI)** |  | **Mean/%** | **(SE)** |  | **OR** | **(95% CI)** |
| Treatment type^a^ |  |  |  |  |  |  |  |  |  |  |  |  |  |  |  |  |  |
| Mental health specialist + psychotherapy | 52.1 | (1.7) |  | 0.66 | (0.42-1.04) |  | 54.9 | (2.3) |  | 0.62* | (0.45-0.85) |  | 55.8 | (2.7) |  | 1.15 | (0.74-1.76) |
| Mental health specialist + medication | 52.0 | (1.9) |  | 1.18 | (0.77-1.81) |  | 60.6 | (2.5) |  | 0.77 | (0.59-1.01) |  | 61.9 | (2.7) |  | 1.56* | (1.08-2.26) |
| General medical | 72.0 | (1.5) |  | 0.59* | (0.36-0.98) |  | 78.8 | (1.6) |  | 0.41* | (0.31-0.55) |  | 82.8 | (1.6) |  | 1.43 | (0.92-2.21) |
| Complementary/alternative medicine | 17.1 | (1.3) |  | 2.19* | (1.29-3.70) |  | 24.0 | (2.2) |  | 0.79 | (0.60-1.05) |  | 25.6 | (2.7) |  | 2.68* | (1.81-3.98) |
| Human services | 14.6 | (1.3) |  | 1.00 | - |  | 19.2 | (2.0) |  | 1.00 | - |  | 21.0 | (2.4) |  | 1.00 | - |
| χ^2^_4_ | 20.23* | | | | |  | 36.90* | | | | |  | 31.10* | | | | |
| Exactly 2 or more of the above | 62.9 | (1.6) |  | 2.04* | (1.17-3.55) |  | 73.6 | (1.7) |  | 1.17 | (0.81-1.71) |  | 75.8 | (1.9) |  | 2.60* | (1.55-4.34) |
| χ^2^_1_ | 6.36* | | | | |  | 0.70 | | | | |  | 13.16* | | | | |
| χ^2^_5_ | 42.83* | | | | |  | 48.15* | | | | |  | 142.93* | | | | |
| Panic disorder or agoraphobia with/without panic | 19.6 | (1.4) |  | 1.71* | (1.10-2.66) |  | 25.2 | (2.4) |  | 0.86 | (0.67-1.11) |  | 26.5 | (2.7) |  | 2.00* | (1.35-2.96) |
| χ^2^_1_ | 5.71* | | | | |  | 1.33 | | | | |  | 12.02* | | | | |
| Childhood adversities |  |  |  |  |  |  |  |  |  |  |  |  |  |  |  |  |  |
| Family dysfunction^b^ | 30.6 | (1.5) |  | 0.64* | (0.44-0.91) |  | 29.9 | (1.8) |  | 0.75* | (0.58-0.95) |  | 30.4 | (2.0) |  | 0.95 | (0.69-1.30) |
| Other^c^ | 16.6 | (1.3) |  | 1.73* | (1.13-2.63) |  | 16.0 | (1.5) |  | 1.12 | (0.82-1.52) |  | 15.2 | (1.6) |  | 1.67* | (1.10-2.54) |
| χ^2^_2_ | 11.50* | | | | |  | 5.87 | | | | |  | 5.80 | | | | |
| Global χ^2^_8_ | 58.43* | | | | |  | 52.88* | | | | |  | 164.79* | | | | |
|  |  |  |  |  |  |  |  |  |  |  |  |  |  |  |  |  |  |

Abbreviations. CI, confidence interval; GAD, generalized anxiety disorder; OR, odds ratio; SE, standard error.

*Significant at .05 level, two-sided test.

^a^Treatment providers: mental health specialists (psychiatrist, psychiatric nurse, psychologist, psychiatric social worker, mental health counselor), primary care providers, human services providers (social

worker or counselor in a social services agency, spiritual advisor), and complementary/alternative medicine (other type of healer or self-help group).

^b^Family dysfunction includes: physical abuse, sexual abuse, neglect, parental mental disorder, parental substance use disorder, parental criminal behavior, and family violence.

^c^Other childhood adversities include: parental death, parental divorce, other loss of a parent, physical illness, and economic adversity.

| **eTable 10. (Started GAD treatment 1990 to 1999): Predictors of obtaining helpful treatment (person-level composite outcome) and of the decomposed encounter-level outcomes of helpful treatment and persistence, among people with lifetime DSM-5 generalized anxiety disorder who obtained treatment** | | | | | | | | | | | | | | | | | |
| --- | --- | --- | --- | --- | --- | --- | --- | --- | --- | --- | --- | --- | --- | --- | --- | --- | --- |
|  |  | | | | |  |  | | | | |  |  | | | | |
|  | **Composite outcome** | | | | |  | **Decomposed outcomes** | | | | | | | | | | |
|  | **Model 1:**  **Person-level relative-odds of**  **obtaining helpful**  **treatment**  **(regardless of the number of professionals seen)** | | | | |  | **Model 2:**  **Encounter-level relative-odds of obtaining helpful treatment from**  **a given professional**  **(pooled across professionals seen)** | | | | |  | **Model 3:**  **Encounter-level relative-odds of the respondent persisting in help-seeking after prior unhelpful treatment**  **(pooled across all unhelpful treatments)** | | | | |
|  | **Prevalence** | |  | **Multivariate** | |  | **Prevalence** | |  | **Multivariate** | |  | **Prevalence** | |  | **Multivariate** | |
|  | **Mean/%** | **(SE)** |  | **OR** | **(95% CI)** |  | **Mean/%** | **(SE)** |  | **OR** | **(95% CI)** |  | **Mean/%** | **(SE)** |  | **OR** | **(95% CI)** |
| Treatment type^a^ |  |  |  |  |  |  |  |  |  |  |  |  |  |  |  |  |  |
| Mental health specialist + psychotherapy | 59.0 | (1.9) |  | 1.16 | (0.73-1.86) |  | 64.8 | (2.2) |  | 0.68* | (0.48-0.94) |  | 66.5 | (2.3) |  | 2.09* | (1.27-3.46) |
| Mental health specialist + medication | 59.9 | (1.9) |  | 1.94* | (1.28-2.96) |  | 70.3 | (2.0) |  | 0.85 | (0.64-1.14) |  | 72.3 | (2.1) |  | 2.70* | (1.85-3.95) |
| General medical | 76.4 | (1.5) |  | 1.32 | (0.85-2.05) |  | 79.8 | (1.6) |  | 0.82 | (0.64-1.04) |  | 80.6 | (1.8) |  | 1.78* | (1.15-2.74) |
| Complementary/alternative medicine | 24.1 | (1.6) |  | 1.19 | (0.74-1.92) |  | 31.1 | (2.1) |  | 0.67* | (0.54-0.83) |  | 33.4 | (2.4) |  | 1.92* | (1.22-3.04) |
| Human services | 18.9 | (1.3) |  | 1.00 | - |  | 22.5 | (1.9) |  | 1.00 | - |  | 23.8 | (2.1) |  | 1.00 | - |
| χ^2^_4_ | 10.05* | | | | |  | 21.79* | | | | |  | 34.14* | | | | |
| Exactly 2 or more of the above | 72.6 | (1.7) |  | 0.65 | (0.37-1.16) |  | 81.1 | (1.6) |  | 0.82 | (0.53-1.29) |  | 83.5 | (1.7) |  | 0.84 | (0.48-1.47) |
| χ^2^_1_ | 2.08 | | | | |  | 0.72 | | | | |  | 0.36 | | | | |
| χ^2^_5_ | 10.53 | | | | |  | 48.16* | | | | |  | 66.97* | | | | |
| Panic disorder or agoraphobia with/without panic | 19.3 | (1.5) |  | 2.09* | (1.33-3.29) |  | 24.5 | (2.2) |  | 0.86 | (0.69-1.08) |  | 25.2 | (2.5) |  | 2.97* | (2.00-4.42) |
| χ^2^_1_ | 10.27* | | | | |  | 1.67 | | | | |  | 28.84* | | | | |
| Childhood adversities |  |  |  |  |  |  |  |  |  |  |  |  |  |  |  |  |  |
| Family dysfunction^b^ | 32.4 | (1.6) |  | 0.84 | (0.59-1.21) |  | 33.5 | (1.8) |  | 1.04 | (0.84-1.29) |  | 33.9 | (2.1) |  | 0.59* | (0.41-0.84) |
| Other^c^ | 19.6 | (1.3) |  | 1.09 | (0.73-1.61) |  | 19.3 | (1.6) |  | 0.89 | (0.73-1.09) |  | 19.2 | (1.7) |  | 1.24 | (0.80-1.91) |
| χ^2^_2_ | 0.93 | | | | |  | 1.36 | | | | |  | 8.97* | | | | |
| Global χ^2^_8_ | 25.51* | | | | |  | 58.95* | | | | |  | 86.21* | | | | |
|  |  |  |  |  |  |  |  |  |  |  |  |  |  |  |  |  |  |

Abbreviations. CI, confidence interval; GAD, generalized anxiety disorder; OR, odds ratio; SE, standard error.

*Significant at .05 level, two-sided test.

^a^Treatment providers: mental health specialists (psychiatrist, psychiatric nurse, psychologist, psychiatric social worker, mental health counselor), primary care providers, human services providers (social

worker or counselor in a social services agency, spiritual advisor), and complementary/alternative medicine (other type of healer or self-help group).

^b^Family dysfunction includes: physical abuse, sexual abuse, neglect, parental mental disorder, parental substance use disorder, parental criminal behavior, and family violence.

^c^Other childhood adversities include: parental death, parental divorce, other loss of a parent, physical illness, and economic adversity
